# Effects of Chinese Mind-Body Exercises on Executive Function in Middle-Aged and Older Adults: A Systematic Review and Meta-Analysis

**DOI:** 10.3389/fpsyg.2021.656141

**Published:** 2021-05-21

**Authors:** Fei-Fei Ren, Feng-Tzu Chen, Wen-Sheng Zhou, Yu-Min Cho, Tsung-Jung Ho, Tsung-Min Hung, Yu-Kai Chang

**Affiliations:** ^1^Department of Physical Education, Beijing Language and Culture University, Beijing, China; ^2^Sport Neuroscience Division, Advanced Research Initiative for Human High Performance, Faculty of Health and Sport Sciences, University of Tsukuba, Ibaraki, Japan; ^3^College of Physical Education, Nanjing Xiaozhuang University, Nanjing, China; ^4^Tzu Chi Medical Foundation, Alhambra, CA, United States; ^5^Integration Center of Traditional Chinese and Modern Medicine, Hualien Tzu Chi Hospital, Hualien, Taiwan; ^6^Department of Chinese Medicine, Hualien Tzu Chi Hospital, Hualien, Taiwan; ^7^Department of Physical Education, National Taiwan Normal University, Taipei, Taiwan; ^8^Institute for Research Excellence in Learning Science, National Taiwan Normal University, Taipei, Taiwan

**Keywords:** aging, cognitive function, cognitive control, physical activity, Tai Chi, Qigong, research synthesis

## Abstract

Chinese mind-body exercises (CMBEs) are positively associated with executive function (EF), but their effects on EF, from synthesized evidence using systematic and meta-analytic reviews, have not been conducted. Therefore, the present systematic review with meta-analysis attempted to determine whether CMBEs affect EF and its sub-domains, as well as how exercise, sample, and study characteristics moderate the causal relationship between CMBEs and EF in middle-aged and older adults. Seven electronic databases were searched for relevant studies published from the inception of each database through June 2020 (PubMed, Web of Science, Embase, Cochrane Controlled Trials Register, Wanfang, China National Knowledge Infrastructure, and Weipu). Randomized controlled trials with at least one outcome measure of CMBEs on EF in adults of mean age ≥ 50 years with intact cognition or mild cognitive impairment (MCI) and with or without chronic diseases were included. A total of 29 studies (*N* = 2,934) ultimately were included in this study. The results indicated that CMBEs improved overall EF (Standardized Mean Differences = 0.28, 95% CI 0.12, 0.44), as well as its sub-domains of working memory and shifting. The beneficial effects of CMBEs on EF occurred regardless of type (Tai Chi, Qigong), frequency of group classes (≤2 time, 3-4 time, ≥5 times), session time (≤45 min, 46-60 min), total training time (≥150 to ≤300 min, >300 min), and length of the CMBEs (4-12 week, 13-26 week, and >26 week), in addition to that more frequent participation in both group classes and home practice sessions (≥5 times per week) resulted in more beneficial effects. The positive effects of CMBEs on EF were also demonstrated, regardless of participants mean age (50-65 years old, >65 years old), sex (only female, both), and cognitive statuses (normal, MCI, not mentioned), health status (with chronic disease, without chronic disease), as well as training mode (group class, group class plus home practice) and study language (English, Chinese). This review thus suggests that CMBEs can be used as an effective method with small to moderate and positive effects in enhancing EF, and that more frequent group classes and home practice sessions may increase these effects. However, certain limitations, including strictly design studies, limited ES (effect size) samples for specific variables, and possible biased publications, required paying particular attention to, for further exploring the effects of CMBEs on EF.

## Introduction

As people grow older, they are susceptible to decline in executive function (EF) (Zaninotto et al., 2018; Caballero et al., 2020). EF is a meta-level and top-down mental process that involves purposeful efforts to supervise, monitor, and control non-automatic behaviors in order to achieve consciously chosen goals (Diamond, 2013). In structural terms, EF can be differentiated into two dimensions, namely, core EF, which in turn involves three distinguishable sub-domains (i.e., inhibition, working memory, and shifting) (Diamond, 2020), and higher-level EF, which involves planning and problem-solving (Romine and Reynolds, 2005). Specifically, inhibition refers to the capacity to deliberately suppress or control a prepotent/dominant stimulus, and to filter out irrelevant cues (Zhan et al., 2020); working memory refers to an ability to hold and manipulate relevant information in the mind temporarily (Miyake et al., 2000); shifting is defined as the capacity to flexibly switch back-and-forth between mental sets, operations or conceptual representations (Kofler et al., 2019); and planning and problem-solving refer to developing an organized approach for accomplishing actions in advance and are aimed at finding out how to reach a specific goal (Mefoh et al., 2017). While consistent decline in both dimensions of EF is observed with aging, the rates and trajectories of those decline are significantly predicted by environmental and lifestyle factors, such as physical activity (Etnier and Chang, 2019; Chen et al., 2020a), fitness level (Bento-Torres et al., 2019), cognitive training (Nguyen et al., 2019), and social interaction and meditation (Chang et al., 2019; Caballero et al., 2020).

Chinese mind-body exercises (CMBEs) are characterized as multi-modal form of exercises that incorporate aerobic exercise, muscular exercise, coordinative exercise, social interaction, and meditation (Chang et al., 2014). CMBEs can be divided into Tai Chi/Tai Chi Chuan and Qigong and seem to be a promising activity for delaying cognitive decline or improving cognitive functions, including memory, attention, cognitive flexibility, and visuospatial perception (Lim et al., 2019; Yang J. et al., 2020). Furthermore, current systematic reviews further demonstrated that CMBEs can be a way to protect against cognitive decline in older adults (Zheng et al., 2015; Zhang et al., 2018). CMBEs also involve mental stimulation through memorizing, concentrating, and meditating during a series of postures, such that CMBEs simultaneously facilitate physical, cognitive, social, and even spiritual functions (Fong et al., 2014; Wayne et al., 2014; Zhang et al., 2018). Studies associated with CMBEs on EF have mainly focused on Tai Chi (Miller and Taylor-Piliae, 2014; Wayne et al., 2014; Chan et al., 2019; Wei et al., 2020). Despite that Tai Chi has shown benefits compared to other single forms of exercise (e.g., aerobic exercise and resistance exercise) (Northey et al., 2018; Chen et al., 2020a), these studies have either focused on an overall EF with a small number of RCT studies (*k* = 4) (Wayne et al., 2014) or a specific EF sub-domain with a limited number of RCT and Non-RCT studies (*k* = 2-5) without considering core EF and higher-level EF in particular (Wu et al., 2013; Zheng et al., 2015). Additionally, some studies have shown that Tai Chi had evidenced no improvements on EF in older adults (Hall et al., 2009; Gerritsen et al., 2020). Such various results suggest that a comprehensive understanding of the effects of CMBEs on EF is still lacking.

The strength of the effects of CMBEs on EF may be influenced by factors related to exercise characteristics, individual background, and study design. The frequency, intensity, time, type, volume, and progression (FITT-VP) of an exercise have been hypothesized as relevant for optimizing the effects of the exercise (ACSM, 2018). Relatedly, Chen et al. (2020a) reported that exercise training interventions with a frequency of 3 to 4 times per week, performed with vigorous intensity, or a total length of training period ranging from 1 to 3 months, demonstrated larger effects on overall EF. However, previous reviews did not focus on specific CMBEs interventions and the moderators, regarding that the effects of FITT-VP for CMBEs are still under investigated. Moreover, individual background factors including age, sex, cognitive status, and health status may also influence the relationship between CMBEs and EF. For example, while Tai Chi has been found to improve EF in healthy adults, it was associated with heterogenous EF outcomes in older adults with early-stage dementia (Lim et al., 2019; Yang J. et al., 2020). Meanwhile, the design of a study (e.g., whether a study includes a control group with active or passive status or includes group classes combined with home practice sessions) may also affect the influences of CMBEs on EF found by the study, such that aspects of study design require further consideration.

In this study, we attempted to fill three research gaps by conducting a systematic review and meta-analysis, targeting randomized controlled trial (RCT) studies examining the effects of CMBEs on EF in middle-aged and older adults with intact cognition and mild cognitive impairment (MCI). In additional to cognitive status, the review also targets on participants with or without chronic diseases. Specifically, our primary aim was to determine whether CMBEs affect EF and its sub-domains of core EF and higher-level EF. Additionally, subgroup analyses of exercise characteristics were performed in order to better understand the dose-related effects of CMBEs interventions. Lastly, analyses were also conducted to determine whether moderators such as individual background factors and study design factors affect the casual relationship between CMBEs and EF.

## Methods

### Design and Eligibility Criteria

This systematic review and meta-analysis was conducted following the Preferred Reporting Items for Systematic Reviews and Meta-Analyses (PRISMA) guidelines (Liberati et al., 2009) and the Cochrane Collaboration Handbook guidelines (Higgins et al., 2019).

Eligible articles were included, according to the following PICOS criteria: *Participants*: studies included adults aged ≥ 50 years who had intact cognition or those diagnosed with MCI. Participants with or without chronic diseases (e.g., traumatic brain injury, Parkinson’s disease, diabetes, depression, stroke) were also included. *Interventions*: only studies involving interventions consisting exclusively of CMBEs (i.e., Tai Chi or Qigong) were included, while studies involving interventions combining Tai Chi or Qigong with other types of interventions (e.g., dance, memory intervention, and transcranial direct current stimulation interventions) were excluded. *Control groups*: studies using either active control group (e.g., physical exercise, educational program, social interaction, cognitive training) or passive control group (e.g., usual care, waitlist control, no intervention) were included. *Outcome*: studies including EF outcomes were examined. *Study design*: only RCT studies were included. Furthermore, eligible articles were published in either peer-reviewed journal in English or consisted of high-quality research (i.e., doctoral dissertations and papers published in Core Chinese periodicals) in Chinese.

### Literature Search

Electronic articles published from the inception of a given database through June of 2020 were searched for in seven databases, namely, the PubMed, Web of Science, Embase, CENTRAL, Wanfang, China National Knowledge Infrastructure, and Weipu databases.

The article searches were conducted by two authors (FFR and FTC), and the search strategy consisted of using medical subject headings (MeSH) for “Tai Ji” OR Qigong AND executive function OR cognition AND randomized control trial (Supplementary 1). After searching for relevant articles, the same two authors (FFR and FFC) first screened the titles and abstracts of potentially eligible articles in accordance with the eligibility criteria. Then, the full-text articles were independently reviewed. If any disagreements occurred between the two authors, the third author (YKC) was consulted until a consensus was reached.

### Data Extraction

The data extraction strategy used was mainly inspired by recent meta-analyses (Northey et al., 2018; Xue et al., 2019; Chen et al., 2020a), and the process of data extraction was conducted according to the Cochrane Collaboration Handbook (Higgins et al., 2019). In this respect, two authors (FFR and FTC) extracted the relevant data from the included studies in a standardized manner.

The data extracted included the following: study (authors, year) and language, participants (age, sex, cognitive/health status), grouping and sample size, CMBE interventions [frequency of group classes (GC), frequency of home practice sessions (HP), session time of GC, length], and EF sub-domain. Furthermore, the following exercise characteristics were calculated: frequency of GC/frequency of group classes plus home practices (GC/GC + HP) and total training time of GC/GC + HP per week.

For each included study, the change of mean (mean_change_) and the standard deviation of change (SD_change_) from pre-test to post-test were extracted. If these values were not available, they were calculated using the following formula: “mean_change_ = mean_post_ - mean_pre_” and “SD_change_ = Square root (SQRT) [(SD _pre_^2^ + SD _post_^2^) – (2 × Corr × SD_pre_ × SD_post_)],” while the correlation coefficient (Corr) was set as 0.5 (Higgins and Green, 2005; Xue et al., 2019). When articles only reported standard errors (SEs) or 95% confidence intervals (CIs), the SD was computed using the formula “SD = SE × SQRT (n) or SD = SQRT (n) × [(upper limit - lower limit)/3.92].” If the change values or mean and *SD*-values of pre-test and post-test were not reported, the sample sizes and *p*-Value were used to calculate the effect size. If any relevant data was missing data, the corresponding author or authors were contacted by the first researcher via email (Higgins et al., 2019).

The overall EF was calculated by using all of the EF outcomes and the EF sub-domains were classified into four areas (core EF: inhibition, working memory, shifting; high-level EF: planning) based upon relevant EF assessments (Table 1).

**TABLE 1:** The assessments and the classification of executive function (EF).

	Core EF		Higher-level EF
Inhibition	Working memory	Shifting	Planning
Auditory Stroop test	Digit Span Backward Test	Chinese Trail Making Test B	Block design Test
Flanker task (Incongruent)	Digit Span forward-backward	Shifting Attention	Clock Drawing Task
Frontal assessment battery	Digit Span longest	Task-switching	
Go/No-go Test	Image Recall	TMT B-A difference	
Stroop Test (color-word)	Rey auditory verbal learning test	Trail Making Test B	
		Trail Making Test B-A	

*The assessments and the classification of executive function (EF) was followed by Romine and Reynolds (2005), Faria et al. (2015), Chen et al. (2020a), and Diamond (2020), respectively.*

With respect to exercise characteristics followed by FITT-VP, six variables were focused on and coded in terms of categories: (1) CMBEs types were coded as Tai Chi and Qigong; (2) frequencies of GC were coded as low (≤2 times), moderate (3-4 times), and high (≥5 times); (3) frequencies of GC/frequencies of GC + HP were also coded as low (≤2 times), moderate (3-4 times), and high (≥5 times); (4) session times of GC were coded as short (≤45 min), moderate (>45 to ≤60 min), and long (>60 min); (5) total training times of GC/GC + HP per week were coded as short (<150 min), moderate (≥150 to ≤300 min), and long (>300 min); and (6) intervention lengths were coded as short (4-12 weeks), moderate (13-26 weeks), and long (>26 weeks). Regarding intensity of CMBEs, most studies did not provide the details, so the variable was not included.

With regard to sample and study characteristics, seven variables were targeted and coded in terms of categories: (1) mean ages were coded as 50-65 years old and >65 years old; (2) sex were coded as female only and both; (3) cognitive statuses were coded as normal, MCI, and not mentioned; (4) health statuses were coded as without chronic disease and with chronic disease; (5) control groups were coded as active control and passive control group; (6) training modes were coded as GC and GC + HP; and (7) languages were coded as English and Chinese.

### Assessment of Study Quality

The quality of the included studies was assessed independently by two authors (FFR and FTC) based on the principles of the Physiotherapy Evidence Database (PEDro). The PEDro scale includes 11 items (eligibility criteria, random allocation, Concealed allocation, similarity baseline, subject blinding, Therapist blinding, Assessor blinding, >85% retention, Intention-to-treat, Between-group comparisons, Point & variability measures), and each study was assessed as either “yes” (score 1) or “no” (score 0) for each of those items. According to the PEDro guidelines, the maximum total score is 10 (item 1 is not used to compute the total score, since it is linked with external validity). If a study receives a score of 9 or 10, it is considered to be of very good quality, while a score of 6 to 8 indicates good quality, a score of 4 or 5 indicates medium quality, and a score of 0 to 3 indicates poor quality (Maher et al., 2003). Any score on which the two authors disagreed was discussed with the third author (YKC) until a consensus was achieved.

### Statistical Analysis

This meta-analysis was performed using Comprehensive Meta-Analysis (CMA) Software, Version 3.0 (Biostat, Englewood, NJ, United States), and the random-effects model was applied based on the assumption of different true effect sizes (Higgins et al., 2019). Random effect model was recommended for meta-analysis with that the true effect sizes were not identical (Kisamore and Brannick, 2008; Schmidt et al., 2009), thus this model was used in the review.

The pooled effect sizes were estimated with standardized mean differences (SMD) and 95% CIs. A positive effect size (ES) indicated that the CMBEs group outperformed the control groups. A *p*-value of 0.05 was regarded as significantly improvement for CMBEs group in comparison to the control groups. The magnitudes of the ESs were classified as small (0.20 to 0.49), moderate (0.50 to 0.79), and large (0.80 to 1.00) (Wilson et al., 2019). The statistical heterogeneity was evaluated using the Q statistic and *I*^2^ values, which range from 0 to 100% (1-49% = small, 50-74% = medium, 75-100% = large, Higgins et al., 2003). In addition, publication bias was assessed by generating a funnel plot and conducting Egger’s regression test.

The steps of the meta-analysis were as follows: (1) the overall effect of CMBEs on the overall EF was calculated. (2) Subgroup analysis was conducted on categorical moderators to examine the effects of CMBEs on EF in terms of three groups, including (1) the EF sub-domains (i.e., inhibition, working memory, shifting, and planning); (2) exercise characteristics (i.e., type, frequency of GC, frequency of GC/GC + HP, session time of GC, total training time of GC/GC + HP per week, and length); (3) sample and study characteristics (age, sex, cognitive status, health status, control groups, training mode, and language).

## Results

### Study Identification and Selection

**FIGURE 1:**
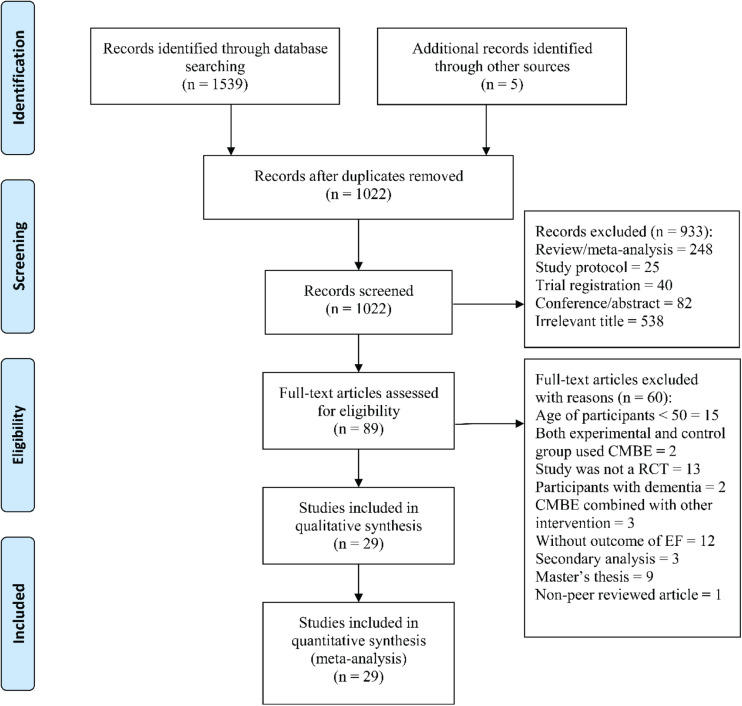
Preferred Reporting Items for Systematic Reviews and Meta-Analyses flow chart of the study identification and selection process. CMBEs, Chinese mind-body exercises; RCT, randomized controlled trial; EF, executive function; *n*, number.

The flow of the study identification and selection process is summarized in Figure 1. The initial search identified 1,544 potentially relevant articles (1,262 articles in English and 282 articles in Chinese). After removing duplicate citations (*k* = 522), 1,022 articles remained for screening according to their titles and abstracts. The screening process left 89 full-text articles, of which 29 RCT articles with a total of 2,934 participants (Table 2), including 26 articles in English (Taylor-Piliae et al., 2010; Lam et al., 2011, 2012; Lavretsky et al., 2011; Mortimer et al., 2012; Nguyen and Kruse, 2012; Sun et al., 2015; Walsh et al., 2015; Lu et al., 2016; Sungkarat et al., 2016, 2018; Chan and Tsang, 2017, 2018; Tao et al., 2017; Myers et al., 2018; Vergara-Diaz et al., 2018; Wu et al., 2018; You et al., 2018; Lipsitz et al., 2019; Riegle van West et al., 2019; Xia et al., 2019; Chewning et al., 2020; Hwang et al., 2020; Moon et al., 2020; Yang Y. et al., 2020; Zheng et al., 2020) and 3 articles in Chinese (Lin, 2016; Li, 2017; Zhao et al., 2020) met the eligibility criteria for qualitative analysis. Data for two articles with missing data were made available upon request (Lavretsky et al., 2011; Riegle van West et al., 2019), and all 29 articles were ultimately included in the quantitative synthesis.

**TABLE 2:** Overview of characteristics associated with Chinese mind-body exercises and executive function studies (*n* = 29).

Study/language	Mean age (years)	Sex	Cognitive/health status	Grouping and sample size	CMBEs interventions	EF task: domain
Chan and Tsang (2017)/Eng.	63.4	Both	Normal/Stroke	I: Modified 12-form Yang-style Tai Chi (*N* = 9) C1: Conventional Exercise (*N* = 8) C2: No intervention (*N* = 9)	GC: 2 times/week, 60 min/session HP: 1 times/week, 30 min/session Length: 12 weeks	AST: Inhibition
Chan and Tsang (2018)/Eng.	62.7	Both	Normal/Stroke	I: Modified Yang-style Tai Chi (*N* = 15) C1: Conventional exercise (*N* = 17) C2: No intervention (*N* = 15)	GC: 2 times/week, 60 min/session HP: 1 times/week, 30 min/session Length: 12 weeks	AST: Inhibition
Chewning et al. (2020)/Eng.	73.9	Both	NR/-	I: Yang-style Tai Chi (*N* = 94) C: Wait-list control (*N* = 103)	GC: 2 times/week, 90 min/session HP: 6 times/week, 25 min/session Length: 6 weeks	TMT-B: Shifting
Hwang et al. (2020)/ Eng.	66.6	Both	Normal/Traumatic brain injury	I: 8-form Yang-style Tai Chi (*N* = 32) C1: computerized cognitive training (*N* = 32) C2: Usual care (*N* = 32)	GC: 1 times/week, 50 min/session HP: 3 times/week, NR min/session Length: 6m	TMT-B: Shifting
Lam et al. (2011)/ Eng.	77.8	Both	MCI/-	I: 24 forms simplified Tai Chi (*N* = 171) C: Stretching and toning (*N* = 218)	GC: 3 times/week, 30 min/session HP: - Length: 5m	DSTB: Working Memory CTMT-B: Shifting
Lam et al. (2012)/Eng.	77.8	Both	MCI/-	I: 24 forms simplified Tai Chi (*N* = 171) C: Stretching and toning (*N* = 218)	GC: 3 times/week, 30 min/session HP: -Length: 52 weeks	DSTB: Working Memory CTMT-B: Shifting
Lavretsky et al. (2011)/Eng.	70.6	Both	Normal/Depression	I: Tai Chi Chih (*N* = 36) C: Health education (*N* = 37)	GC: 1 times/week, 120 min/session HP: -Length: 10 weeks	SCWT: Inhibition TMT-B: Shifting
Li (2017)/Chi.	65.8	Both	MCI/Hyperlipemia, Hypertension	I: Qigong (Baduanjin) (*N* = 45) C1: Brisk walking (*N* = 45) C2: Health education (*N* = 45)	GC: 3 times/week, 60 min/session HP: -Length: 24 weeks	GNGT: Inhibition TMT-B: Shifting
Lin (2016)/Chi.	67.3	Both	MCI/-	I: Qigong (Baduanjin) (*N* = 49) C: Health education (*N* = 49)	GC: 7 times/week, 60 min/session HP: -Length: 24 weeks	IR: Working memory
Lipsitz et al. (2019)/Eng.	75.3	Both	MCI/Hypertension, depression, diabetes, cancer	I: Yang-style Tai Chi (*N* = 93) C: Health education (*N* = 87)	GC: 2 times/week, 60 min/session HP: 1 times/week, 20 min/session Length: 6m	TMT-B-A: Shifting
Lu et al. (2016)/Eng.	70.1	Female	Normal/-	I: 12-form Yang style Tai Chi (*N* = 15) C: General interest classes (*N* = 16)	GC: 3 times/week, 90 min/session HP: -Length: 16 weeks	AST: Inhibition
Moon et al. (2020)/Eng.	66.2	Both	Normal/Parkinson’s disease	I: Qigong (six healing sounds) (*N* = 8) C: Sham Qigong (*N* = 9)	GC: 3 times/week + 1 times/week, 45-60 min/session HP: 14 times/week, 15-20 min/session Length: 3 weeks + 9 weeks	FAB: Inhibition TMT-B: Shifting
Mortimer et al. (2012)/Eng.	67.8	Both	Normal/-	I: Tai Chi (*N* = 30) C1: Walking (*N* = 30) C2: Social interaction (*N* = 30) C3: No intervention (*N* = 30)	GC: 3 times/week, 50 min/session HP: -Length: 40 weeks	SCWT: Inhibition DSTB: Working Memory TMT-B: Shifting CDT: Planning
Myers et al. (2018)/Eng.	53.7	Female	NR/Breast cancer, diabetic	I: Qigong (six healing sounds) (*N* = 19) C1: Gentle exercise (*N* = 20) C2: Attention control (*N* = 11)	GC: 1 times/week, 60 min/session HP: 2 times/week, 15 min/session Length: 8 weeks	RAVLT: Working memory TMT-B: Shifting
Nguyen and Kruse (2012)/Eng.	69	Both	MCI/-	I: Tai Chi (*N* = 48) C: (No intervention) (*N* = 48)	GC: 2 times/week, 60 min/session HP: -Length: 6m	TMT-B: Shifting
Riegle van West et al. (2019)/Eng.	68.4	Both	NR/-	I: Yang style Tai Chi (*N* = 46) C: Poi (*N* = 50)	GC: 2 times/week, 60 min/session HP: -Length: 4 weeks	SCWT: Inhibition SA: Shifting
Sun et al. (2015)/Eng.	69.2	Both	Normal/Cardiac, hyperpiesia, diabetes mellitus	I: 24-form Yang style Tai Chi (*N* = 72) C: playing cards or singing (*N* = 66)	GC: 2 times/week, 60 min/session HP: -Length: 6m	FAB: Inhibition
Sungkarat et al. (2016)/Eng.	67.9	Both	MCI/-	I: 10-form Tai Chi (*N* = 33) C: No intervention (*N* = 33)	GC: 3 times/week, 50 min/session HP: 3 times/week, 50 min/session Length: 15 weeks	DSTFB: Working memory TMT-B-A: Shifting BDT: Planning
Sungkarat et al. (2018)/Eng.	67.9	Both	MCI/-	I: 10-form Tai Chi (*N* = 33) C: No intervention (*N* = 33)	GC: 3 times/week, 50 min/session HP: 3 times/week, 50 min/session Length: 6m and 3 weeks	DSTFB: Working memory TMT-B-A: Shifting BDT: Planning
Tao et al. (2017)/Eng.	61.6	Both	Normal/-	I1: Yang style 24-form Tai Chi (*N* = 21) I2:Qigong (Baduanjin) (*N* = 16) C: No intervention (*N* = 24)	GC: 5 times/week, 60 min/session HP: -Length: 12 weeks	DSTFB: Working memory
Taylor-Piliae et al. (2010)/Eng.	69.1	Both	NR/Hypertension, angina, asthma	I: Yang short-form Tai Chi (*N* = 37) C1: Western Exercise (*N* = 39) C2: Healthy aging class (*N* = 56)	GC: 1-2 times/week, 45 min/session HP: 3 times/week, 45 min/session Length: 52 weeks	DSTB: Working memory
Vergara-Diaz et al. (2018)/Eng.	63.9	Both	NR/Parkinson’s disease	I: Tai Chi (*N* = 16) C: No intervention (*N* = 16)	GC: 2 times/week, 60 min/session HP: NR times/week, 75 min/session Length: 6m	TMT-B: Shifting
Walsh et al. (2015)/Eng.	64.2	Both	Normal/Hypertension	I: Yang, Wu, Chen, or Sun style Tai Chi (*N* = 31) C: usual care (*N* = 29)	GC: 2 times/week, 30 min/session HP: 2 times/week, 30 min/session Length: 6m	DSLB: Working memory TMT-B: Shifting
Wu et al. (2018)/Eng.	64.9	Both	Normal/-	I: 24-form Yang-style Tai Chi (*N* = 16) C: No intervention (*N* = 15)	GC: 3 times/week, 60 min/session HP: -Length: 12 weeks	TS: Shifting
Xia et al. (2019)/Eng.	65.5	Both	MCI/-	I: Qigong (Baduanjin) (*N* = 23) C1: Brisk walking (*N* = 23) C2: Usual physical activity (*N* = 23)	GC: 3 times/week, 60 min/session HP: -Length: 24 weeks	SCWT: Inhibition
Yang Y. et al. (2020)/Eng.	66.1	Female	Normal/-	I: 8-form Yang-style Tai Chi (*N* = 13) C: Usual care (*N* = 13)	GC: 3 times/week, 45 min/session HP: -Length: 8 weeks	FT: Inhibition
You et al. (2018)/Eng.	74.5	Both	Normal/Chronic multisite pain, diabetes	I: Tai Chi (*N* = 22) C: light physical exercise (*N* = 23)	GC: 2 times/week, 60 min/session HP: 1 times/week, NR min/session Length: 12 weeks	TMT-B: Shifting
Zhao et al. (2020)/Chi.	64	Both	NR/-	I: 24 forms simplified Tai Chi (*N* = 60) C: Usual care (*N* = 60)	GC: 5 times/week, 60 min/session HP: -Length: 12 weeks	SCWT: Inhibition
Zheng et al. (2020)/Eng.	62.1	Both	MCI/Stroke	I: Qigong (Baduanjin) + Health education (*N* = 24) C: Health education (*N* = 24)	GC: 3 times/week + 1 times/m, 40 min/session HP: - Length: 24 weeks	GNGT: Inhibition TMT-B: Shifting CDT: Planning

*Eng. = English; Chi. = Chinese; yrs = years; both = male and female; *N* = number; I = intervention; C = control group; MCI = mild cognitive impairment; GC = group class; HP = home practice; wk = week; wks = weeks; m = month; min = minute; - = no; NR = not report; EF = executive function; CMBEs interventions = Chinese mind-body exercise interventions; AST = Auditory stroop task; TMT-B = Trail making test B; DSTB = Digit span test backward; CTMT-B = Chinese trail making test B; SCWT = Stroop color-word test; GNGT = Go/No-go task; IR = Image recall; TMT-B-A = Trail making test B-A difference; FAB = Frontal assessment battery; CDT = Clock drawing task; RAVLT = Rey auditory verbal learning test; SA = Shifting attention; DSTFB = Digit span test forward-backward; BDT = Block design test; DSLB = Digit span longest backward; TS = Task switching; FT = Flanker task (Incongruent).*

### Characteristics of Included Studies

With respect to the sample characteristics of the included studies, the mean ages of the participants ranged from 53.7 to 77.8 years old. Among the included articles (*k* = 29), 26 investigated both male and female participants and 3 focused exclusively on female subjects. With regard to cognitive status, 13 studies targeted participants with intact (normal) cognitive status, 10 studies for MCI, and 6 studies does not provide the details. We also found that 14 of the studies targeted participants with chronic diseases (e.g., traumatic brain injury, Parkinson’s disease, diabetes, depression, and stroke).

With regard to exercise characteristics, 23 studies focused on Tai Chi and 7 studies focused on Qigong. In terms of control groups, 19 studies used an active control group and 14 studies used a passive control group. The frequency of the CMBEs investigated in the studies ranged from 1 to 7 times. The session time for group classes varied from 30 minutes to 120 minutes. The overall length of the interventions ranged from 4 weeks to 52 weeks. Furthermore, 13 studies used group classes plus home practice sessions for their interventions and 16 studies used group classes only.

With respect to EF sub-domains, 19 studies examined shifting as the EF outcome. In addition, 13 studies targeted inhibition and 10 studies targeted working memory. The review found that there were only 4 studies that assessed the planning aspect of EF.

The quality assessment results of the included studies are shown in Table 3. The results indicated that 20 of the studies received scores of 6-8 (good quality), 7 studies received scores of 4-5 (median quality), and two studies received scores of 3 (poor quality). Overall, the mean quality of the included studies was scored as 6.

**TABLE 3:** Quality assessment of included studies (*n* = 29).

	Eligibility	Random	Concealed	Similarity	Subject	Therapist	Assessor	>85%	Intention	Between-group	Point & variability	Total
	criteria	allocation	allocation	baseline	blinding	blinding	blinding	retention	-to-treat	comparisons	measures	score
Chan and Tsang (2017)	1	1	0	1	0	0	1	0	1	1	1	6
Chan and Tsang (2018)	1	1	0	1	0	0	1	1	1	1	1	7
Chewning et al. (2020)	1	1	0	0	0	0	0	0	0	1	1	3
Hwang et al. (2020)	1	1	0	0	0	0	1	0	1	1	1	5
Lam et al. (2011)	1	1	0	1	0	0	1	0	1	1	1	6
Lam et al. (2012)	1	1	0	1	0	0	1	0	1	1	1	6
Lavretsky et al. (2011)	1	1	1	1	0	0	1	1	1	1	1	8
Li (2017)	1	1	1	0	0	0	1	0	0	1	1	5
Lin (2016)	1	1	1	1	0	0	0	1	1	1	1	7
Lipsitz et al. (2019)	1	1	0	1	0	0	1	0	1	1	1	6
Lu et al. (2016)	1	1	0	0	0	0	1	1	1	1	1	6
Moon et al. (2020)	1	1	1	1	1	0	1	0	0	1	1	7
Mortimer et al. (2012)	1	1	0	1	0	0	0	1	1	1	1	6
Myers et al. (2018)	1	1	0	0	0	0	0	1	1	1	1	5
Nguyen and Kruse (2012)	1	1	0	1	0	0	0	0	0	1	1	4
Riegle van West et al. (2019)	1	1	0	0	0	0	1	0	0	1	0	3
Sun et al. (2015)	1	1	1	1	0	0	0	1	1	1	1	7
Sungkarat et al. (2016)	1	1	1	1	0	0	1	1	1	1	1	8
Sungkarat et al. (2018)	1	1	1	1	0	0	1	1	1	1	1	8
Tao et al. (2017)	1	1	0	1	0	0	1	0	0	1	1	5
Taylor-Piliae et al. (2010)	1	1	1	1	0	0	1	0	1	1	1	7
Vergara-Diaz et al. (2018)	1	1	0	1	0	0	1	0	0	1	1	5
Walsh et al. (2015)	1	1	1	1	0	0	0	1	1	1	1	7
Wu et al. (2018)	1	1	0	1	0	0	1	1	1	1	1	7
Xia et al. (2019)	1	1	0	1	0	0	0	1	0	1	1	5
Yang Y. et al. (2020)	1	1	0	1	0	0	1	1	0	1	1	6
You et al. (2018)	1	1	1	0	0	0	1	0	1	1	1	6
Zhao et al. (2020)	1	1	0	1	0	0	0	1	1	1	1	6
Zheng et al. (2020)	1	1	1	1	0	0	1	1	1	1	1	8
Mean score												6

### Overall Analysis, Heterogeneity, and Small Sample Size Bias

**FIGURE 2:**
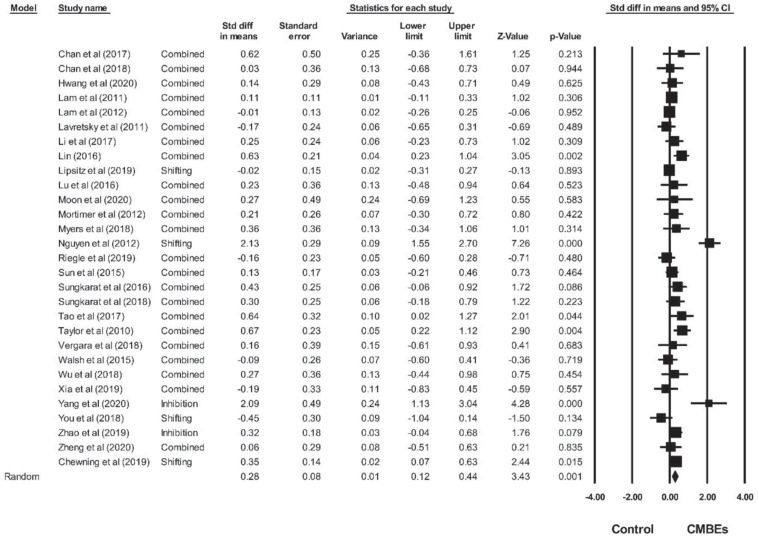
Forest plot of Chinese mind-body exercises on overall EF.

**FIGURE 3:**
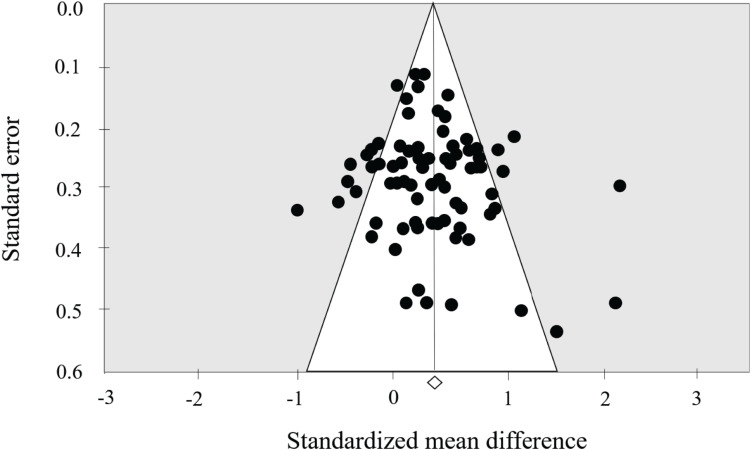
Funnel plot of publication bias.

The results indicated significant improvement in overall EF after the CMBEs interventions in the participating older adults. The pooled ES for CMBEs was significant and small ES (SMD = 0.28, 95% CI 0.12 to 0.44, *p* = 0.001), with medium heterogeneity (*I*^2^ = 68.96%, *p* < 0.001) compared to the control groups (Figure 2). Regarding publication bias, visual inspection of the funnel plot showed that it was symmetrical, and the results further showed a non-significant Egger’s regression intercept (*t* = 1.50, *p* = 0.14) revealing the absence of funnel plot asymmetry (Figure 3).

### Subgroup Analysis

The results of the subgroup analyses are summarized in Table 4 and described below. To ensure the most beneficial effect on EF, we performed the subgroup analyses to examine the moderating roles of different factors on the effects of CMBEs on EF, with the analyses including EF sub-domains, as well as exercise, sample, and study characteristics.

**TABLE 4:** Analysis and subgroup analysis results regarding the effects of Chinese mind-body exercises on executive function.

Subgroup analysis	k	SMD (95% CI)	*p*-value	Test of heterogeneity		
				*I*^2^%	Q	*p*-value
**EF dimension**					0.33	0.954
Inhibition	13	0.18 (−0.04, 0.39)	0.107	47.23		
Working memory	10	0.22 (0.03, 0.40)	0.021	47.51		
Shifting	19	0.26 (0.05, 0.47)	0.015	76.09		
Planning	4	0.20 (−0.05, 0.46)	0.120	0		
**Exercise characteristics**						
Type					0.25	0.618
Tai Chi	23	0.24 (0.13, 0.35)	<0.001	62.34		
Qigong	7	0.19 (0.04, 0.35)	0.01	62.69		
Frequency (GC)					4.05	0.132
Low (≤2 time)	15	0.23 (0.08, 0.38)	0.003	69.52		
Moderate (3-4 time)	11	0.19 (0.07, 0.30)	0.002	55.69		
High (≥5 times)	3	0.56 (0.21, 0.91)	0.002	44.20		
Frequency (GC + HP)					8.13	0.017
Low (≤2 time)	5	0.19 (−0.06, 0.44)	0.145	85.73		
Moderate (3-4 time)	18	0.17 (0.08, 0.27)	0.001	50.94		
High (≥5 time)	6	0.54 (0.31, 0.77)	<0.001	10.06		
Session time (GC)					0.80	0.671
Short (≤45 min)	6	0.20 (0.01, 0.38)	0.036	61.44		
Moderate (46-60 min)	20	0.25 (0.14, 0.36)	<0.001	63.59		
Long (>60 min)	3	0.09 (−0.27, 0.46)	0.612	39.28		
Total training time (GC + HP)					4.09	0.129
Short (<150 min)	13	0.13 (−0.01, 0.27)	0.065	68.95		
Moderate (≥150, ≤300 min)	13	0.27 (0.15, 0.38)	<0.001	51.58		
Long (>300 min)	3	0.47 (0.11, 0.83)	0.01	42.98		
Length					0.03	0.986
Short (4-12 week)	12	0.23 (0.07, 0.39)	0.005	45.86		
Moderate (13-26 week)	13	0.23 (0.10, 0.37)	0.001	73.95		
Long (>26 week)	4	0.21 (0.03, 0.40)	0.025	46.20		
**Sample and study characteristics**						
Age					1.00	0.316
50-65 (years)	9	0.17 (0.04, 0.31)	0.011	51.40		
>65 (years)	20	0.27 (0.15, 0.39)	<0.001	69.08		
Sex					2.48	0.115
Only female	3	0.51 (0.15, 0.87)	0.006	65.37		
Both	26	0.21 (0.12, 0.30)	<0.001	61.44		
Cognitive status					0.91	0.633
Normal	13	0.20 (0.06, 0.34)	0.006	45.03		
MCI	10	0.22 (0.08, 0.35)	0.001	73.60		
Not mentioned	6	0.33 (0.10, 0.56)	0.005	48.45		
Health status					1.27	0.270
Without chronic disease	15	0.28 (0.15, 0.40)	<0.001	72.31		
With chronic disease	14	0.18 (0.05, 0.30)	0.006	45.08		
Control groups					5.63	0.018
Active group	19	0.16 (0.05, 0.26)	0.003	56.61		
Passive group	14	0.39 (0.23, 0.55)	<0.001	66.22		
Training mode					0.53	0.468
GC	16	0.20 (0.09, 0.31)	<0.001	69.58		
GC + HP	13	0.27 (0.12, 0.43)	0.001	33.76		
Language					0.91	0.339
English	26	0.21 (0.11, 0.30)	<0.001	60.22		
Chinese	3	0.33 (0.10, 0.56)	0.005	69.82		

*k, number of included studies; *I*^2^, *I* square; GC, group class; HP, home practice; EF, executive function; MCI, mild cognitive impairment; yrs, years; CI, confidence interval; SMD, standardized mean differences.*

### EF Sub-Domains

**FIGURE 4:**
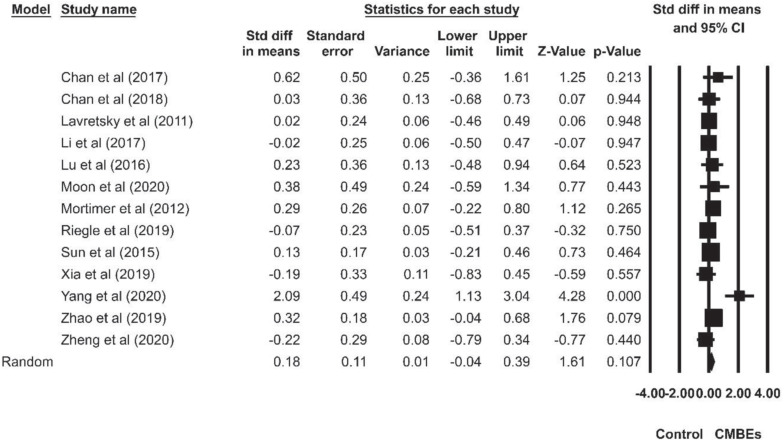
Forest plot of Chinese mind-body exercises on inhibition.

**FIGURE 5:**
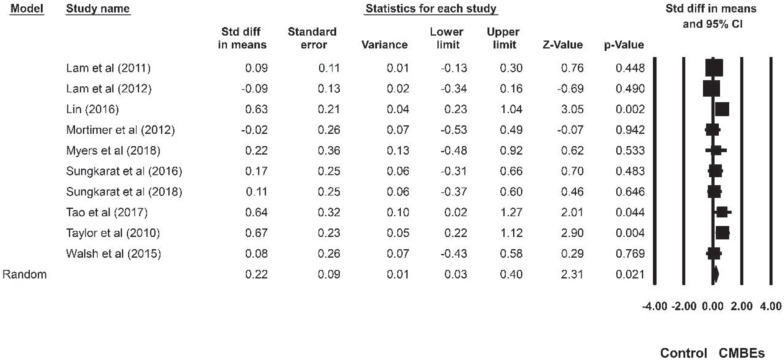
Forest plot of Chinese mind-body exercises on working memory.

**FIGURE 6:**
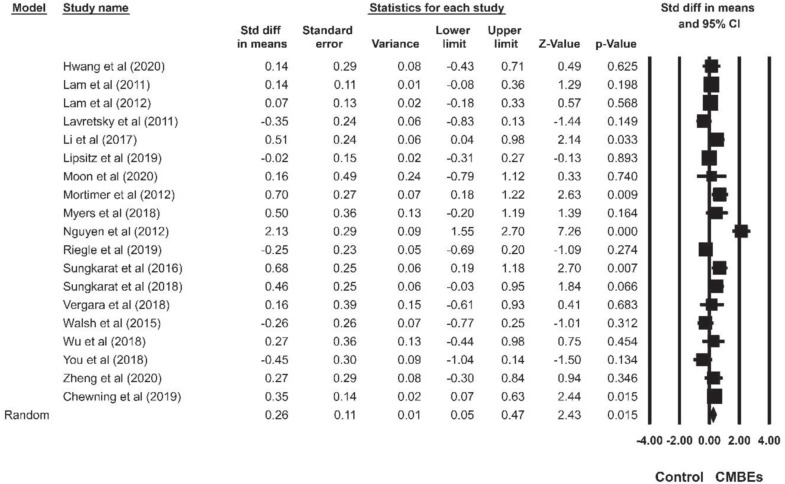
Forest plot of Chinese mind-body exercises on shifting.

**FIGURE 7:**
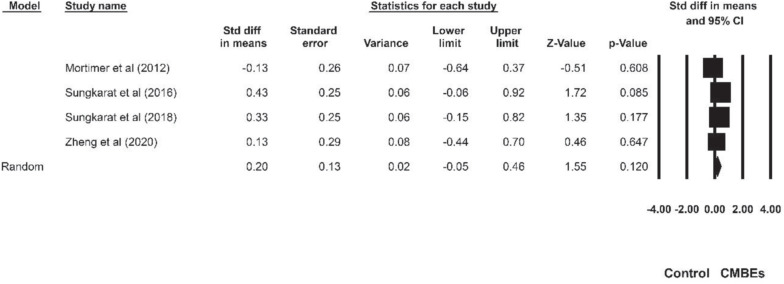
Forest plot of Chinese mind-body exercises on planning.

The results indicated no significant differences among the four sub-domains of EF after the CMBEs interventions (*p* = 0.954) (Table 4). Regarding the core EF, there was no significant ES for inhibition (SMD = 0.18, *p* = 0.107) compared to the control groups (Figure 4). Additionally, the results indicated that there were significant and small ESs for both working memory (SMD = 0.22, *p* = 0.021) and shifting (SMD = 0.26, *p* = 0.015) compared to the control groups (Figures 5, 6). Regarding the higher-level EF, the results showed no significant ES for planning (SMD = 0.20, *p* = 0.120) compared to the control groups (Figure 7).

### Exercise Characteristics

Regarding exercise type, there was no significant difference between the two types of CMBEs (*p* = 0.618), and the results showed significant and small ESs for both Tai Chi (SMD = 0.24, *p* < 0.001) and Qigong interventions (SMD = 0.19, *p* = 0.01) compared to the control groups.

With regard to frequency, the present study focused on the frequency of GC and the frequency of GC/GC + HP. Regarding frequency of GC, the results indicated that there was no significant difference among the three variables (*p* = 0.132), revealing a significant and moderate ES for high frequency (SMD = 0.56, *p* = 0.002), significant and small ESs for both low frequency (SMD = 0.23, *p* = 0.003) and moderate frequency (SMD = 0.19, *p* = 0.002) interventions as compared to the control groups. Regarding the frequency of GC/GC + HP, the results indicated that effect of CMBEs on EF was significantly influenced by the frequency of GC + HP (*p* = 0.017). The results revealed that there was a significant and moderate ES for high frequency (SMD = 0.54, *p* < 0.001) and a significant and small ES for moderate frequency (SMD = 0.17, *p* = 0.001), but a non-significant ES for low frequency (SMD = 0.19, *p* = 0.145) compared to the control groups.

With respect to session time and total training time, the results showed no significant difference among the three session time ranges (*p* = 0.671). The results indicated significant and small ESs for both short session times (≤45 min) (SMD = 0.20, *p* = 0.036) and moderate session times (46-60 min) (SMD = 0.25, *p* < 0.001) compared to the control groups. In contrast, there was no significant ES for long session times (>60 min) (SMD = 0.09, *p* = 0.612). The results indicated no significant difference among the three ranges of total training time per week (*p* = 0.129). The results indicated significant and small ESs for both long total training times (>300 min) (SMD = 0.47, *p* = 0.01) and moderate total training times (≥150, ≤300 min) (SMD = 0.27, *p* < 0.001), but a non-significant ES for short total training times (<150 min) (SMD = 0.13, *p* = 0.065) compared to the control groups.

Regarding CMBEs length, there was no significant difference among the three ranges of length (*p* = 0.986). The results revealed significant and small ESs for short lengths (4-12 weeks) (SMD = 0.23, *p* = 0.005) and moderate lengths (13-26 weeks) (SMD = 0.23, *p* = 0.001), as well as for long lengths (>26 weeks) (SMD = 0.21, *p* = 0.025) compared to the control groups.

### Sample and Study Characteristics

With regard to mean age, there was no significant difference between individuals aged 50-65 years old and individuals aged > 65 years old (*p* = 0.316). The results showed significant and small ESs for individuals aged > 65 years old (SMD = 0.27, *p* < 0.001) and for individuals aged 50-65 years old (SMD = 0.17, *p* = 0.011) compared to the control groups. In terms of sex, there was no significant difference between the studies that only included female participants and the studies that included both male and female participants (*p* = 0.115). The results indicated a significant and moderate ES for studies with female participants alone (SMD = 0.51, *p* = 0.006) and a significant and small ES for studies with participants of both sex (SMD = 0.21, *p* < 0.001) compared to the control groups.

Regarding cognitive status, the results indicated there was no significant difference among the three variables of cognitive status (*p* = 0.633). The results revealed significant and small ESs for participants with normal cognition (SMD = 0.20, *p* = 0.006), for those with MCI (SMD = 0.22, *p* = 0.001), and for the participants whose cognitive status was not mentioned (SMD = 0.33, *p* = 0.005) compared to the control groups. With regard to health status, the results indicated that there was no significant difference between the different health statuses (*p* = 0.270). The results showed significant and small ESs for participants without chronic disease (SMD = 0.28, *p* < 0.001) and participants with chronic disease (SMD = 0.18, *p* = 0.006) compared to the control groups.

In terms of control groups, the results indicated that the effects of CMBEs on EF were significantly influenced by control groups (*p* = 0.018). Specifically, although the ESs were significant and small, the ES when comparing a CMBEs group with a passive control group (SMD = 0.39, *p* < 0.001) was greater than the ES when comparing a CMBEs group with an active control group (SMD = 0.16, *p* = 0.003). With regard to training mode, the results showed that there was no significant difference between training modes (*p* = 0.468), revealing that the ESs were significant and small ESs for both GC (SMD = 0.20, *p* < 0.001) and GC + HP (SMD = 0.27, *p* = 0.001) compared to the control groups. With respect to language, the results indicated that there was no significant difference between English and Chinese studies (*p* = 0.339), The results showed significant and small ESs for the included studies written in English (SMD = 0.21, *p* < 0.001) and in Chinese (SMD = 0.33, *p* = 0.005) compared to the control groups.

## Discussion

The present systematic review and meta-analysis, for the first time, combined these two forms of Chinese mind-body exercise (Tai Chi and Qigong) and examined the effects of CMBEs interventions on overall EF and its sub-domains in middle-aged and older adults aged ≥ 50 years, while also considering diverse moderators in terms of EF, as well as exercise, sample, and study characteristics. 29 studies with a total of 2,934 participants were analyzed. The review results indicated that CMBEs significantly improved overall EF and its sub-domains of working memory and shifting with significant and small ES. Additionally, the positive effect of CMBEs on EF was found to be influenced by the frequency of group classes or group classes plus home practice sessions, as well as by the control groups selected for exercise and study characteristics, respectively.

### Overall EF

The observed positive effect of CMBEs on overall EF in middle-aged and older adults was not only consistent with but also extends previous meta-analyses that targeted adults with MCI (Zou et al., 2019), those focused on a variety of cognitive functions (Wayne et al., 2014; Chan et al., 2019), those that investigated west-east mind-body exercise (Chan et al., 2019), and those that examined exercise in broad terms (Northey et al., 2018; Chen et al., 2020a).

The beneficial effect of CMBEs on EF may be the result of in the multi-modal nature of CMBEs, that is, its inclusion of “cardiovascular fitness, motor fitness, movement coordination, social interaction, and meditation” (Chang et al., 2014). These features have been demonstrated to be associated with structural and functional changes of the brain in middle-aged and older adults (Wei et al., 2013; Fong et al., 2014; Chen et al., 2020b), especially in the prefrontal cortex, temporal cortex, hippocampus, and medial prefrontal cortex (mPFC), all of which play important roles in EF (Tao et al., 2016; Wu et al., 2018). Additionally, CMBEs have been observed to induce the production of brain-derived neurotrophic factor (BDNF), which is able to stimulate cerebrovascular regeneration, synaptic plasticity, and cell proliferation in the hippocampus and frontal cortex (Mortimer et al., 2012; Sungkarat et al., 2018; Audiffren and André, 2019; Marinus et al., 2019).

### EF Sub-Domains

The present meta-analysis is among the first to examine whether EF sub-domains influence the effects of CMBEs on EF. Our finding of non-significant differences between core EF and higher-level EF implies that the EF improvements derived from CMBEs occur regardless of EF sub-domains. The finding expands current review conducted by Chen et al. (2020a), observing similar effects on EF sub-domains from exercise in general (e.g., aerobic exercise, resistance exercise, and Tai Chi), and suggesting that Tai Chi had larger effects than other types of exercise.

In particular, the present review found that there were beneficial effects of CMBEs on the working memory and shifting aspect of the core EF, which are critical for the activities of daily living (Diamond, 2013). The results were consistent with previous findings, suggesting that mind-body exercises (MBEs) (i.e., Tai Chi, Yoga, dance) resulted in positive improvements of such EF sub-domains (Wu et al., 2019). Furthermore, Chen et al. (2020a) also observed similar effects on working memory and shifting from exercises and further suggested that MBEs (i.e., Tai Chi and Yoga) had more moderate ES than other types of exercise. Despite studies showing significant improvements in working memory and shifting, however, we emphasized that only 3 out of 10 included studies for working memory (Taylor-Piliae et al., 2010; Lin, 2016; Tao et al., 2017), and only 4 out of 19 included studies for shifting (Mortimer et al., 2012; Nguyen and Kruse, 2012; Sungkarat et al., 2016; Li, 2017), with moderate to high study quality showing positive ES on EF. Notably, although studies below moderate study quality also have shown positive ES for working memory and shifting, the possible bias of their methods may have led to confounded results between the CMBEs and the control groups. Following the results of the study quality assessment, it is worth noting that three items (i.e., concealed allocation, subject blinding, therapist blinding) are mainly concerns leading to questions over how such studies were judged as being below moderate study quality, and consequently, we thus suggest that future studies should pay close attention to conducting studies with strictly monitored design, that might then reduce possible biases, in order to investigate the effects of CMBE on EF.

Despite finding no adverse effects of CMBEs, our review did observe a negligible ES on the inhibition and planning aspect of EF. We surprised there was a non-significant ES on the inhibition, which is inconsistent with previous findings focusing on exercise training (Chen et al., 2020a). It is worth noting, however, that the number of included studies for inhibition (*k* = 13) or planning (*k* = 4) was not enough to draw a conclusion, implying that the observed effects of CMBEs on inhibition and planning should be carefully interpreted. Indeed, more studies examining inhibition and planning have been called for in order to further our understanding of exercise effects on EF comprehensively. Therefore, future research should consider multiple EF assessments, so as to investigate more fully the effects of CMBEs on EF (Etnier and Chang, 2009; Faria et al., 2015).

### Exercise Characteristics

This review investigated six CMBEs characteristics as moderators in order to determine the optimal dose of CMBEs for improving EF. Regarding CMBEs type, the subgroup analysis showed no difference in positive EF effects between Tai Chi and Qigong. This finding supports previous meta-analysis results suggesting improvements from Tai Chi (Wayne et al., 2014), whereas it was inconsistent with past reports indicating no effects on EF from Qigong (Chan et al., 2019). This contradiction might have resulted from the number of included study, where only one study of Qigong was included in a past report (Chan et al., 2019), while 7 RCT studies (*k* = 7) were included in our review.

This review also found no difference in terms of the frequency of group classes, implying that CMBEs interventions undertaken 1 to 5 times per week have a positive effect on EF. However, we observed that interventions with greater frequency (≥5 times per week) had effects twice as large as those with low frequency (≤2 times per week) and moderate frequency (3-4 times per week) in terms of combined group classes and home practice session, implying that greater exercise frequencies, particularly in terms of practice sessions at home, provide better effects. This review also considered three duration variables (i.e., session time, total training time, and length) as potential moderators but observed no significant differences. While a majority of the investigated studies applied session times of 46-60 min, total training times per week of 150 to 300 min, and lengths between 13 and 26 weeks, our findings suggest that doses of more or less than these duration variables still demonstrate positive effects, which provides a lower barrier for engaging in CMBEs in the older adults.

### Sample and Study Characteristics

The present meta-analysis evaluated four sample characteristic variables (i.e., age, sex, cognitive status, and health status), and none of these variables showed a moderating effect. The similar positive EF effects from CMBEs for different ages (i.e., 50-65 vs. >65 years old) as well as sex (i.e., female and both sex) indicated that the EF benefits of CMBEs can be available regardless of age or sex. This review also provides encouraging information updating the conclusions of previous studies that the beneficial EF effects associated with CMBEs can also be observed in older adults with or without cognitive impairment (i.e., those with normal cognition or MCI) or with good or poor health (i.e., those with or without chronic disease) (Northey et al., 2018; Chan et al., 2019; Zou et al., 2019; Chen et al., 2020a).

Three variables of study design (i.e., control groups, training mode, and language) were considered as moderators, and the significant moderating effect found for control groups requires more attention. Specifically, CMBEs had greater ES (SMD = 0.39) when comparing with passive control group (i.e., usual care, waitlist control, no intervention) than ES when comparing with active control group (SMD = 0.16) (i.e., physical exercise, educational program, social interaction, cognitive training). Furthermore, these findings were consistent with those of past studies examining exercise mode as a moderator, in which Tai Chi was found to have larger EF effects than aerobic exercise, resistance exercise, and even other types of mind-body exercise (Northey et al., 2018; Chen et al., 2020a). It is possible that this is because Tai Chi has a multi-modal nature that provides more effects, although this topic warrants further examination (Chang et al., 2014; Lim et al., 2019; Yang et al., 2019; Wei et al., 2020).

### Strengths and Limitations

The present review, the first of its kind regarding RCT studies, was conducted to determine the effects of CMBEs on EF and its sub-domains among middle-aged and older adults. Additionally, moderators associated with general characteristics (e.g., FITT-V and sample background) and new factors, including the frequency of both group classes and home practice sessions, control groups, and language, were further examined, providing a more comprehensive view of the effects of CMBEs on EF than past reviews. However, the present review also had several limitations. Firstly, only a doctoral dissertation and 2 journal papers of high quality were eligible to be included from the investigated Chinese databases, a factor that may have led to a language bias. While our initial thought was to include only high-quality studies, the issue of eligibility based upon language may need further consideration. Secondly, given the limited outcomes linkage several variables [e.g., planning (EF sub-domain), high frequency (frequency), long session times (time), only female (sex), Chinese (language)], we thus elucidated the results with caution and suggested that more studies will be called for in the future. Thirdly, although the result of an Egger’s test indicated no publication bias in this meta-analysis, the funnel plot was suggestive of biased studies. We could not completely rule out the possibility of publication bias, therefore, the result of this meta-analysis needs to be interpreted cautiously. Last but not least, the limited number of investigated studies regarding higher-level EF suggests the need for further explanation of this sub-domain in the future.

## Conclusion

The present review suggest CMBEs would enhance EF with small positive effects in terms of enhanced EF, including its overall status and sub-domains (i.e., working memory, shifting). The beneficial effects of CMBEs on EF were found regardless of intervention type, the frequency of group classes, session time, total training time, and length of intervention. Additionally, when the frequency of group classes plus home practice sessions was more than 5 times per week, the intervention had a larger effect than when the frequency was less than 4 times per week. The beneficial effects of CMBEs on EF were also demonstrated regardless of participant age, sex, and cognitive and health status, as well as training mode and study language, with CMBEs interventions having additional effects compared to other types of interventions.

## Data Availability Statement

All datasets generated for this research are included in this published article/Supplementary Material.

## Author Contributions

Y-KC, T-MH, F-FR, and F-TC contributed to the conception of the work. Y-KC, T-MH, F-FR, F-TC, W-SZ, Y-MC, and T-JH contributed to the design of the work. F-FR, F-TC, and Y-KC conducted the literature search, selection, data extraction, and analysis. F-FR, F-TC, W-SZ, and Y-KC conducted the assessment of study quality. F-FR, Y-KC, and F-TC wrote the first draft of the manuscript with support from T-MH. All authors contributed to the manuscript revisions and agreed with final approval of the version and ensured the accuracy of research.

## Conflict of Interest

The authors declare that the research was conducted in the absence of any commercial or financial relationships that could be construed as a potential conflict of interest.
