# Supplementary material for: Effects of Chinese Mind-Body Exercises on Executive Function in Middle-Aged and Older Adults: A Systematic Review and Meta-Analysis
Source: Front Psychol. 2021 May 21;12:656141. doi: 10.3389/fpsyg.2021.656141 (PMC8175659; doi:10.3389/fpsyg.2021.656141)
Supplement: Supplementary file 1 [file Table_1.docx]

**SUPPLEMENTARY 1** Search terms used to identify the included studies of Chinese mind-body exercises and executive function

| **Tai Chi** | **Qigong** | **Executive Function** | **Cognition** | **Randomized controlled trial** |
| --- | --- | --- | --- | --- |
| Tai Ji  Tai-ji  Tai Chi  Chi, Tai  Tai Ji Quan  Ji Quan, Tai  Quan, Tai Ji  Taiji  Taijiquan  T'ai Chi  Tai Chi Chuan | Qigong  health Qigong  health Qi Gong  Ch'i Kung  Qigong  qi gong  Chi Kung  Chi Gong  Yijinjing  YiJin Jing  yi jin jing  Yi Jin Jing  Yi-Jin-Jing  yijin-jing  Wuqinxi  Wu Qin Xi  WuQinxi  Liuzijue  Liu Zi Jue  LIU Zi-jue  Baduanjin  BaDuanJin  Ba Duan Jin | Executive function  Executive functions  Function, executive  Functions, executive  Executive control  Executive controls  Cognitive control  Cognitive Controls  Inhibition  Inhibitory Control  Updating  Working memory  Switching  Shifting  Planning  Cognitive Flexibility  Problem solving  Problem-solving  Reasoning | Cognition  Cognitions  Cognitive Function  Cognitive Functions  Function, Cognitive  Functions, Cognitive  Cognitive Performance  Metacognition  Metacognitive Control  Metacognitive Controls | Randomized controlled trial Randomized  Randomised  Random  Randomly  Randomness  Randomization  Control Clinical trial  Controlled Clinical trial  Randomized clinical trial. |

| PubMed search [strategy](javascript:;): |
| --- |
| (((("Tai Ji"[Mesh]) OR (((((((((((Tai Ji[Title/Abstract]) OR (Tai-ji[Title/Abstract])) OR (Tai Chi[Title/Abstract])) OR (Chi, Tai[Title/Abstract])) OR (Tai Ji Quan[Title/Abstract])) OR (Ji Quan, Tai[Title/Abstract])) OR (Quan, Tai Ji[Title/Abstract])) OR (Taiji[Title/Abstract])) OR (Taijiquan[Title/Abstract])) OR (T'ai Chi[Title/Abstract])) OR (Tai Chi Chuan[Title/Abstract]))) OR (("Qigong"[Mesh]) OR (((((((((((((((((((((((Qigong[Title/Abstract]) OR (health Qigong[Title/Abstract])) OR (health Qi Gong[Title/Abstract])) OR (Ch'i Kung[Title/Abstract])) OR (qigong[Title/Abstract])) OR (qi gong[Title/Abstract])) OR (Chi Kung[Title/Abstract])) OR (Chi Gong[Title/Abstract])) OR (Yijinjing[Title/Abstract])) OR (YiJin Jing[Title/Abstract])) OR (yi jin jing[Title/Abstract])) OR (Yi Jin Jing[Title/Abstract])) OR (Yi-Jin-Jing[Title/Abstract])) OR (yijin-jing[Title/Abstract])) OR (Wuqinxi[Title/Abstract])) OR (Wu Qin Xi[Title/Abstract])) OR (WuQinxi[Title/Abstract])) OR (Liuzijue[Title/Abstract])) OR (Liu Zi Jue[Title/Abstract])) OR (LIU Zi-jue[Title/Abstract])) OR (Baduanjin[Title/Abstract])) OR (BaDuanJin[Title/Abstract])) OR (Ba Duan Jin[Title/Abstract])))) AND ((("Executive Function"[Mesh]) OR (((((((((((((((((((executive Function[Title/Abstract]) OR (executive functions[Title/Abstract])) OR (function, executive[Title/Abstract])) OR (functions, executive[Title/Abstract])) OR (executive control[Title/Abstract])) OR (executive controls[Title/Abstract])) OR (cognitive control[Title/Abstract])) OR (cognitive controls[Title/Abstract])) OR (Inhibition[Title/Abstract])) OR (inhibitory control[Title/Abstract])) OR (Updating[Title/Abstract])) OR (working memory[Title/Abstract])) OR (Switching[Title/Abstract])) OR (shifting[Title/Abstract])) OR (Planning[Title/Abstract])) OR (cognitive flexibility[Title/Abstract])) OR (problem solving[Title/Abstract])) OR (problem-solving[Title/Abstract])) OR (reasoning[Title/Abstract]))) OR (("Cognition"[Mesh]) OR ((((((((((Cognition[Title/Abstract]) OR (Cognitions[Title/Abstract])) OR (Cognitive Function[Title/Abstract])) OR (Cognitive Functions[Title/Abstract])) OR (Function, Cognitive[Title/Abstract])) OR (Functions, Cognitive[Title/Abstract])) OR (Cognitive Performance[Title/Abstract])) OR (metacognition[Title/Abstract])) OR (Metacognitive Control[Title/Abstract])) OR (Metacognitive Controls[Title/Abstract]))))) AND ((randomized controlled trial[Publication Type]) OR ((((((((((randomized controlled trial[Title/Abstract]) OR (randomized[Title/Abstract])) OR (randomised[Title/Abstract])) OR (random[Title/Abstract])) OR (randomly[Title/Abstract])) OR (randomness[Title/Abstract])) OR (Randomization[Title/Abstract])) OR (control clinical trial[Title/Abstract])) OR (controlled clinical trial[Title/Abstract])) OR (randomized clinical trial[Title/Abstract]))) |
